# Diagnostic Accuracy of Videofluoroscopy for Symptomatic Cervical Spine Injury Following Whiplash Trauma

**DOI:** 10.3390/ijerph17051693

**Published:** 2020-03-05

**Authors:** Michael D. Freeman, Evan A. Katz, Scott L. Rosa, Bryan G. Gatterman, Ellen M. F. Strömmer, Wendy M. Leith

**Affiliations:** 1 CAPHRI School for Public Health and Primary Care, Faculty of Health, Medicine, and Life Sciences, Maastricht University, 6211 LM Maastricht, The Netherlands; e.m.f.strommer@gmail.com (E.M.F.S.); wendyleith@gmail.com (W.M.L.); 2 Private practice, Boulder, CO 80302, USA; chirokatz@hotmail.com; 3 Private practice, Rock Hill, NY 12775, USA; drscottrosa@hvc.rr.com; 4 Life Chiropractic College West, Hayward, CA 94545, USA; gatterman@comcast.net

**Keywords:** whiplash, instability, videofluoroscopy, digital motion x-ray, positive predictive value

## Abstract

Background: Intervertebral instability is a relatively common finding among patients with chronic neck pain after whiplash trauma. Videofluoroscopy (VF) of the cervical spine is a potentially sensitive diagnostic tool for evaluating instability, as it offers the ability to examine relative intervertebral movement over time, and across the entire continuum of voluntary movement of the patient. At the present time, there are no studies of the diagnostic accuracy of VF for discriminating between injured and uninjured populations. Methods: Symptomatic (injured) study subjects were recruited from consecutive patients with chronic (>6 weeks) post-whiplash pain presenting to medical and chiropractic offices equipped with VF facilities. Asymptomatic (uninjured) volunteers were recruited from family and friends of patients. An ethical review and oversight were provided by the Spinal Injury Foundation, Broomfield, CO. Three statistical models were utilized to assess the sensitivity, specificity, positive and negative predictive values (PPV and NPV) of positive VF findings to correctly discriminate between injured and uninjured subjects. Results: A total of 196 subjects (119 injured, 77 uninjured) were included in the study. All three statistical models demonstrated high levels of sensitivity and specificity (i.e., receiver operating characteristic (ROC) values of 0.71 to 0.95), however, the model with the greatest practical clinical utility was based on the number of abnormal VF findings. For 2+ abnormal VF findings, the ROC was 0.88 (93% sensitivity, 79% specificity) and the PPV and NPV were both 88%. The highest PPV (1.0) was observed with 4+ abnormal findings. Conclusions: Videofluoroscopic examination of the cervical spine provides a high degree of diagnostic accuracy for the identification of vertebral instability in patients with chronic pain stemming from whiplash trauma.

## 1. Introduction

Neck pain is a highly prevalent condition, occurring in 10–21% of the adult population annually [1]. A frequent cause of both acute and chronic neck pain is injury from a motor vehicle crash (MVC) [2]. Although a variety of spinal injuries are associated with MVCs, the most common injury type is musculoligamentous sprain or strain [3]. Such injuries often result from the type of whiplash trauma that is closely associated with rear impact crashes. The term “whiplash” refers to a traumatic whipping motion of the head and neck, primarily occurring in rear impact crashes, that produces higher peak acceleration at the head than in the neck or thoracolumbar spine [4]. While some authors have also used “whiplash” as a generic catch-all description for a variety of injuries resulting from whiplash trauma, when used to describe an injury the term generally refers to cervical spine sprain/strain injury [5]. 

A well-established feature of whiplash trauma is injury to the ligaments of the cervical spine, resulting in joint laxity and instability [6,7]. Intervertebral instability associated with ligamentous injury can be both difficult to detect and refractory to treatment [8]. Because the diagnosis of vertebral “instability” refers to an abnormality of function, the condition is typically not identifiable from static postural radiographs and may be occult to other conventional imaging (i.e., MRI and CT) and thus is prone to underdiagnosis [9]. 

A fluoroscopic examination of the spine (also known as videofluoroscopy (VF) or digital motion x-ray (DMX)) allows for a continuous and minute examination of movement within the cervical spine, including abnormalities of intervertebral motion associated with ligamentous instability [10]. Standard VF records 30 images per second of continuous x-ray of active range of motion across multiple planes, allowing for a dynamic four-dimensional visualization of the integrity of the ligaments of the upper, mid, and lower cervical spine [10,11]. Typically, cervical spine VF motion studies include a lateral view of flexion and extension (to examine anterior to posterior intervertebral instability) and anterior to posterior views of bilateral flexion with the mouth closed (to evaluate for excessive facet gapping in the mid and lower cervical spine) and the mouth open (to evaluate for lateral instability of C1 on C2). The studies provide evidence of the functional integrity of the ligamentum flavum, anterior and posterior longitudinal, interspinous, supraspinous and facet capsular ligaments in the mid and lower cervical spine (C2-7), and the alar and transverse ligaments in the upper cervical spine (C0-2) [7,8,12]. 

Although prior authors have examined the interrater reliability of VF for detecting cervical spine instability [13], at the present time there are no published studies describing the diagnostic accuracy (e.g., positive and negative predictive values) of VF for detecting symptomatic whiplash trauma-associated instability. The goal of the present study is therefore to provide a quantitative assessment of the ability of VF to discriminate between patients with symptomatic post-traumatic neck pain versus asymptomatic controls. 

## 2. Methods

The study population was drawn from consecutive patients and patient relatives or acquaintances at 11 chiropractic or medical offices with an available on-site VF facility. Ethical oversight and approval was provided by a non-profit institutional review board (Spinal Injury Foundation-IRB00002637) in Broomfield, CO, registered with the US Department of Health and Human Services. Verbal informed consent was deemed adequate due to minimal risk, and was obtained from all study subjects in accordance with the IRB approved protocol. The inclusion criteria for all subjects was an age of between 16 and 65 years and an absent history of cervical fracture, congenital anomaly, inflammatory arthritis, diagnosed connective tissue disorders, metastatic disease of the spine, or any other bony or neurological abnormality that was deemed to potentially affect the results of a VF examination. Symptomatic (injured) subjects were recruited from patients actively seeking treatment for subacute or chronic neck pain persisting for more than 6 weeks after a traffic crash-related acute neck injury. Asymptomatic (uninjured) patients were recruited from relatives or acquaintances of patients presenting to the offices, and who did not, in the prior year, have a history of either chronic neck pain or episodic neck pain persisting for >1 week.

There were 5 VF motion view examinations of the cervical spine included in the study: 1) an anterior to posterior (A-P) view of the entire cervical spine, with right and left lateral flexion to the comfort of the patient; 2) A-P view of the upper cervical spine with the mouth open, with right and left lateral flexion to the comfort of the patient; and 3) lateral view with flexion and extension to the comfort of the patient, and 4–5) right and left oblique views with flexion and extension to the comfort of the patient. The limit of translation (slippage) used as the expected threshold for “normal” spines was 2 mm based on the so-called “rule of 2s” [14], as fewer than 10% of the spines of the uninjured and asymptomatic study group would be expected to exceed this degree of slip [15,16]. The upper threshold of normal intervertebral flexion used for the study was 10 degrees [17]. Other parameters used for assessing the VF studies are described in further detail in Table 1.

**Figure 1:**
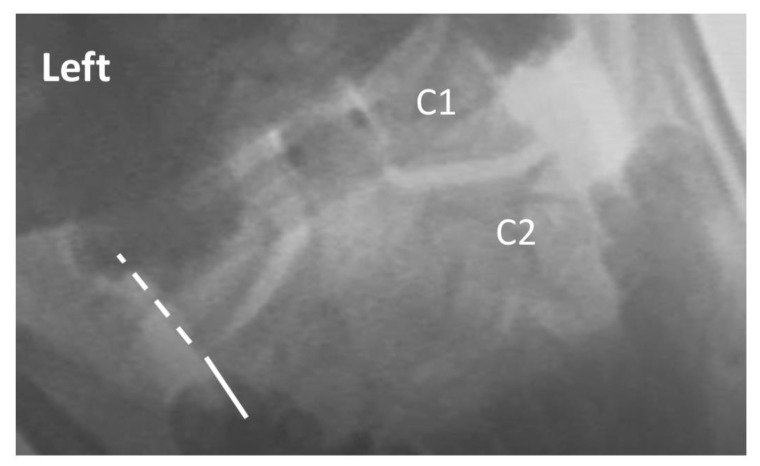
Exemplar of AP open mouth left lateral flexion view of C1 on C2, demonstrating normal alignment. The dashed line indicates the lateral border of the left lateral mass of C1, and the solid line indicates the lateral border of the left articular pillar of C2. The 2 lines are aligned, indicating no translation of C1 on C2 with maximal voluntary left lateral flexion. Note: the image has been reversed so that left on the image corresponds with the patient’s left.

**Figure 2:**
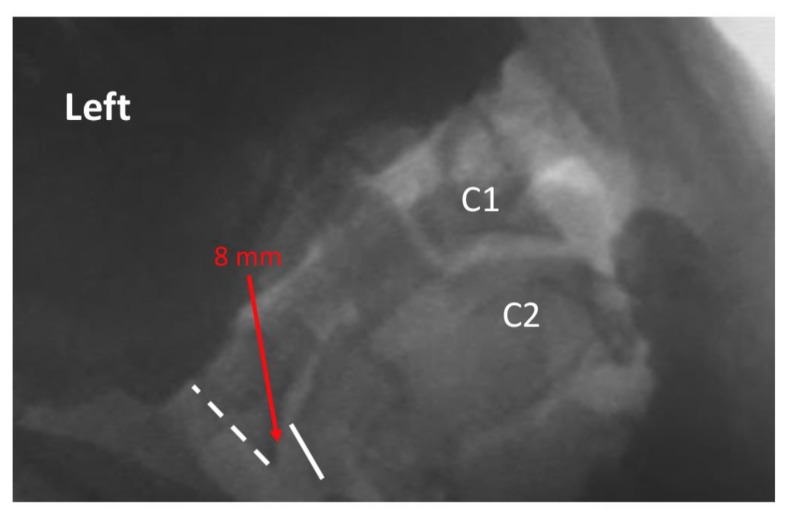
Exemplar of AP open mouth left lateral flexion view of C1 on C2, demonstrating abnormal alignment. The dashed line indicates the lateral border of the left lateral mass of C1, and the solid line indicates the lateral border of the left articular pillar of C2. The red arrow indicates 8 mm lateral translation of C1 on C2 during maximal voluntary lateral flexion. Note: the image has been reversed so that left on the image corresponds with the patient’s left.

**Figure 3:**
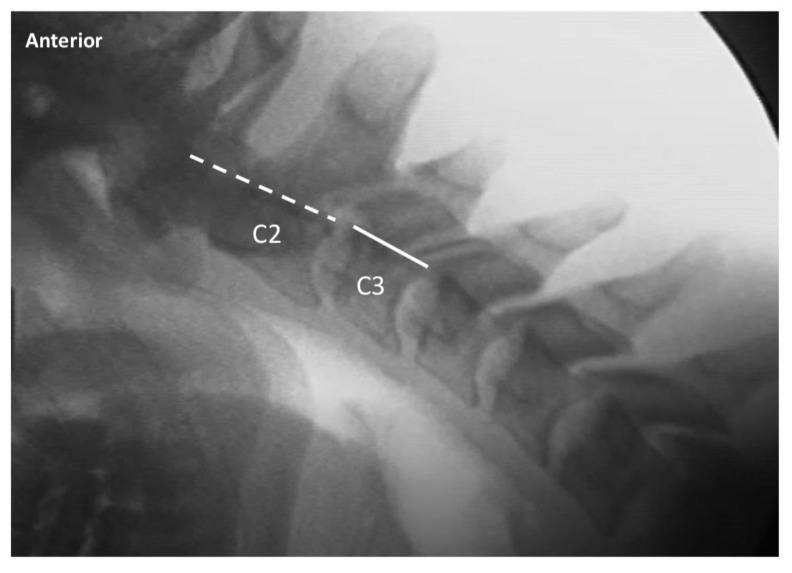
Exemplar of lateral cervical flexion view, demonstrating normal alignment of C2 on C3. The dashed line indicates the posterior margin of the vertebral body of C2, and the solid line indicates the posterior margin of the vertebral body of C3. The 2 lines are aligned, indicating normal alignment of C2 on C3 upon maximal voluntary flexion.

**Figure 4:**
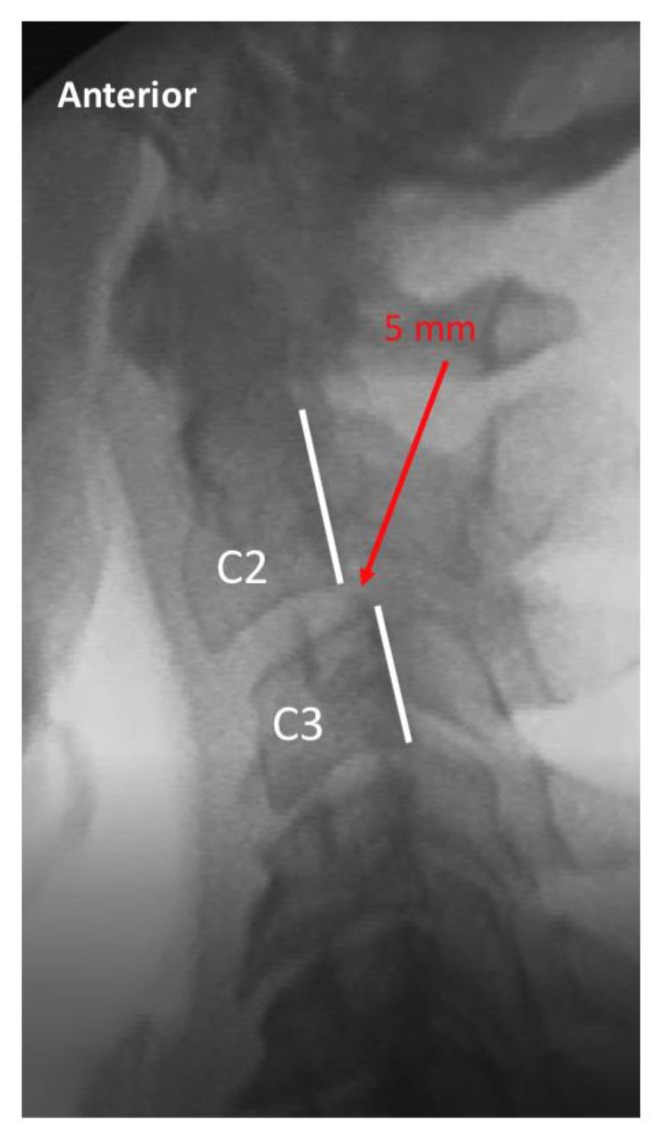
Exemplar of lateral cervical flexion view, demonstrating abnormal alignment of C2 on C3. The dashed line indicates the posterior margin of the vertebral body of C2, and the solid line indicates the posterior margin of the vertebral body of C3. The red arrow indicates 5 mm of anterior translation of C2 on C3 upon maximal voluntary flexion, which is limited due to pain.

Two raters trained in radiology and experienced in interpreting VF examinations evaluated the 5 studies of each patient for intervertebral movement from C1–C7. The raters were blinded as to prior interpretation of the studies. Details of measurement/assessment category choices for the VF readers are listed in Table 2. Where the raters disagreed, the more conservative (normal) of the 2 ratings was used to reduce the risk of Type I (false positive) error. The ratings were then dichotomized into either the “expected normal” finding or not (i.e., abnormal), as detailed in Table 2. Examples of normal and abnormal VF findings are depicted in Figure 1, Figure 2, Figure 3 and Figure 4.

The data were first evaluated for differences between the symptomatic and asymptomatic patients with respect to age, sex, and abnormal VF findings (see Table 3). The differences in average age and total number of abnormal VF findings were evaluated with t-tests, while the categorical variables were evaluated with chi-square tests. The data were then randomly split into a 75% training data set and a 25% testing data set. The training data set was used to build 3 different statistical models to assess the best model for identifying injured patients. The first model was based on combinations of VF measures, age, and sex as established by stepwise logistic regression (entry *p*-value = 0.20; exit *p*-value = 0.05); the second model used the total number of abnormal readings as a continuous variable in an adjusted logistic regression model including age and sex; and the third model was a cut-point analysis to establish a threshold for the dichotomous classification of the total number of abnormal readings. The training model predictive value was quantified with the area under the receiver operating characteristic curve (ROC), while the lack of fit was evaluated with the Hosmer–Lemeshow goodness-of-fit test. The testing data set was used to verify the predictive ability of the models by examining the sensitivity (probability that the test would correctly identify injured patients) and specificity (probability that the test would correctly identify uninjured volunteers). Positive predictive value (PPV) and negative predictive value (NPV) were also calculated for the models. PPV quantifies the probability that an individual with a positive test is an injured patient, and NPV is the probability an individual with a negative test is an uninjured volunteer. All analyses were performed using SAS Software, Version 9.4 (SAS Institute Inc., Cary, NC, USA). The dataset used for the analysis can be accessed in the supplementary Tables S1 and S2.

## 3. Results

A total of 196 subjects were recruited for inclusion in the study, divided into 77 (39.3%) asymptomatic/uninjured volunteers and 119 (60.7%) symptomatic/ injured patients (Table 3). The symptomatic patients were significantly more likely to be female (75.6% vs 46.8%; *p*-value < 0.0001) and were significantly older (40.5 vs 33.9; *p* = 0.0006). 

The uninjured volunteers had substantially fewer abnormal VF readings across all 37 combinations of measures and spinal levels (average 7.0 vs 1.2 per patient; *p* < 0.0001); none of the uninjured volunteers had abnormal findings for 17 of the measured parameters, and only three of the parameters were positive for abnormality in >10% of the asymptomatic group (C1-2 overhang, and C3-4 and C4-5 translation). In comparison, among the injured patients there was at least one subject with abnormality in all of the 37 VF parameters, and in 23 VF parameters, more than 10% of the injured patients were interpreted as abnormal. There were 24 of the VF parameters in which there was a statistically significant difference between the two study groups; in all cases, the frequency among the injured patients was greater than among the uninjured volunteers. See Table 3 for more details.

Three predictive statistical models were examined in order to determine the model with the highest degree of diagnostic accuracy for differentiating between injured patients and uninjured volunteers. These models and their results were as follows:

### 3.1. Model 1

Stepwise logistic regression using all VF parameters, as well as age and sex, was used. Entry *p*-value = 0.20. Exit *p*-value = 0.05. 

Model 1 results: The only single VF parameter found to be a significant predictor for symptomatic patient status was C4-C5 facet gapping. The ROC was 0.71, and the likelihood that a patient with an abnormal C4–C5 facet gapping finding would be symptomatic was 44.9 times greater than a patient with a normal C4–C5 facet gapping finding (95% CI (5.9, 339.3)). The sensitivity and NPV were 0.53 and 0.58, respectively, while the specificity and PPV were both 1 (See Table 4). The lack of fit could not be assessed. 

### 3.2. Model 2 

Logistic regression using the total number of abnormal findings as a continuous measure was used and adjusted for age and sex. 

Model 2 results: The total number of abnormal findings resulted in a model with a ROC of 0.94, indicating near-perfect prediction in the training data. The odds that the subject was injured increased by 2.6 for each additional abnormal finding (95% CI (1.79, 3.69)). Neither age nor sex were significant after accounting for the number of abnormal findings. The sensitivity and PPV were both 0.93, while the specificity and NPV were 0.89 (see Table 4). The model did not suffer from lack of fit. 

### 3.3. Model 3

Cut-point analysis examining the predictive ability of a dichotomization of the number of abnormal findings across a range of values (two or more abnormal findings (2+), three or more abnormal findings (3+), etc.) was used and adjusted for age and sex. 

**Figure 5:**
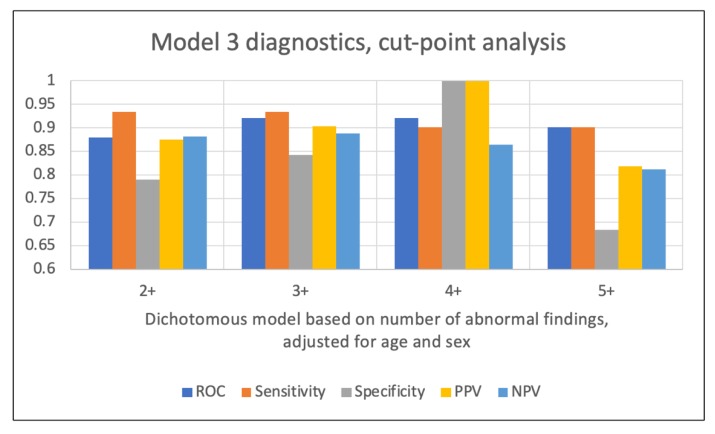
Diagnostic accuracy results for Model 3.

Model 3 results: The ROC increased from 0.88 for the model dichotomized at two or more abnormal findings, to 0.92 for the 3+ and 4+ models, and then decreased for the model dichotomized at five or more abnormal findings. The sensitivity attained a maximum value for the 2+ and 3+ models (0.93), while the specificity was maximized for the 4+ model (see Table 4). PPV hit a maximum (1) in the 4+ model, while NPV was maximized (0.89) in the 3+ model. None of the cut-point models with the exception of the 5+ dichotomization suffered from lack of fit. A comparison of the diagnostics for each of the models is illustrated in Figure 5.

## 4. Discussion

These results provide convincing evidence for several conclusions: (1) intervertebral instability is a *common* finding in the symptomatic population of patients with chronic neck pain after whiplash trauma; (2) intervertebral instability is an *uncommon* finding in the uninjured population; (3) the finding of two or more abnormal parameters of intervertebral motion during the videofluoroscopic examination of the cervical spine is a highly accurate diagnostic test for identifying patients with chronic neck pain after whiplash trauma. 

The findings in the present study demonstrate a common pathological entity (ligamentous laxity) in the population of patients with chronic pain after whiplash, and one that can be identified with a relatively common diagnostic examination. These findings make sense given the fact that injury to the spinal ligaments, and particularly those of the facet capsule, is readily explained from the known pathomechanics of whiplash trauma, in which focal intersegmental hyperextension and hyperflexion have the potential to produce excessive strain (stretch) of the intervertebral ligaments [18]. 

These findings have importance given a number of prior publications demonstrating equivocal or negative MRI results in patients with acute and chronic whiplash, a body of literature that has been transformed into a myth that whiplash is a “soft-tissue” injury that cannot be detected with medical imaging [19]. The repetition of this myth has, in turn, resulted in a number of non-organic, and pejorative explanations for chronic pain complaints after whiplash trauma, including symptom exaggeration, malingering, and secondary gain [20,21]. Given the high prevalence of ligament injury in the chronic whiplash population, and the lack of such findings in the uninjured population, the attribution of complaints to a nonorganic source in any chronic whiplash patient without first ruling out pathology via VF imaging of the cervical spine is unfounded.

A finding in the present study that required further investigation was the three VF parameters that occurred at a higher than expected rate (>10%) in the uninjured versus injured group: the C1-2 lateral overhang difference (32.5% vs. 63.6%), C3-4 translation (11% vs. 11.7%), and C4-5 translation (24.7% vs. 48.7%), respectively. The most direct explanation for these findings is that, for these measures, the cut-point of >2 mm translation (difference or absolute measure) for “abnormal” was too low. A re-examination of the data using 4 mm and below as the cut-point for “normal” demonstrated that this was indeed the case. For the C1-2 lateral overhang, the frequency of abnormal findings among the uninjured volunteers decreased to 5.2%, whereas 34.2% of the injured group were still categorized as abnormal. At C3-4, the increase in the normal cut point to 4 mm or less eliminated all but one of the abnormal translation findings (and the only remaining abnormality was found in the uninjured group). At C4-5, the increased cut point eliminated all of the abnormal findings in the uninjured group and decreased the frequency of abnormal findings in the injured group to 10.5%. These results indicate that the universal reliance on the “rule of 2’s” in all circumstances may increase the risk of false positives in VF for individual measures. This caveat does not apply to the statistical models described in the present study; the high PPV probabilities associated with 2+, 3+, and 4+ positive findings were all based on the 2+ mm threshold for abnormality (for the measured parameters).

All of the examinations in the study were performed using videofluoroscopic equipment that is specifically designed for spinal motion examination at relatively low radiation dosage (Digital Motion X-Ray^®^, Palm Harbor, FL, USA). Because the radiation is pulsed, rather than constant stream technology, and only uses a 2-3 kilovoltage peak (kVp) versus the 80 kVp used for a typical plain cervical x-ray, the total radiation dose for a 5 VF motion study is approximately equivalent to the dose used for a 7 view cervical Davis series.

Although ligaments are not visualized on VF, it is reasonable to infer that a finding of excessive intervertebral movement on VF examination is demonstrative of ligamentous injury when the associated symptoms began shortly after exposure to a whiplash injury mechanism. While some ligament injuries are detectable on MRI, including complete tears, the type of stretching injury that may result in abnormal VF findings are not necessarily correlated with any CT or MRI abnormality, even though the pathology will be evident upon microscopic examination [22]. An intriguing follow-up study would be a “look back” analysis of MRI studies of chronic post-whiplash patients with subsequently positive VF examination.

A caveat to keep in mind when interpreting these results and applying them to the general clinical population is that interrater agreement (Cohen’s kappa) between the two experienced VF readers ranged from only moderate (> 0.4–0.6) for the injured patients to good (>0.6–0.8) for the uninjured volunteers. The impact of this interrater variability on the study results was negated by the use of the most conservative (i.e., normal) of the two ratings when the raters disagreed, as the approach had the effect of decreasing the number of differences between the injured and uninjured study subjects. The use of the less conservative interpretation would have primarily had the effect of increasing the average number of abnormal findings in the injured group, and thus slightly increased the ROC and PPV values in the 2+ and 3+ abnormal findings in the model. Regardless, as with many medical imaging modalities, potential variability in the interpretation of VF studies should be recognized in assessing the results reported by a single reader.

## 5. Conclusions

The videofluoroscopic (or DMX) examination of the cervical spine is a highly accurate test for identifying patients with symptomatic ligamentous instability after whiplash trauma. The imaging modality should be utilized more widely in the clinical investigation of chronic post-whiplash pain.

## Figures and Tables

**Table 1:** Measured parameters of videofluoroscopy (VF) views/motions.

Anatomical/Biomechanical Parameter (View)	Vertebral Level	VF motion Examination/View Details	Measurement Details	Expected Normal Values
Lateral overhang (AP)	C1-2	A-P open mouth, R and L lateral flexion	Maximum difference in lateral translation (in mm) of the lateral margin of the lateral mass of C1 relative to the lateral margin of the superior articular facet of C2, between sides	2 mm or less overhang difference between sides
Peri-odontoid space symmetry (AP)	C1-2	A-P open mouth, R and L lateral flexion	Bilateral symmetry of gap between dens of C2 and medial margin of lateral mass of C1, observed at extreme R and L flexion	Symmetrical gap maintained
Translation (lat)	C2-7	Lateral flexion-extension	Maximum anterior (in flexion) or posterior (in extension) translation of vertebral body relative to adjacent inferior vertebra, measured at posterior vertebral body line	2 mm or less anterior or posterior translation
Intervertebral angulation (lat)	C2-7	Lateral flexion-extension	Angle between adjacent posterior vertebral body lines in maximum flexion	10 degrees or less
SP engagement (lat)	C2-7	Lateral flexion-extension	Degree of synchronous movement between adjacent spinous processes during flexion from neutral	Inter-spinous process distance increases commensurately with flexion
SP coupled movement (AP)	C2-7	AP c-spine R and L lateral flexion	Degree of coupled spinous process rotation with ipsilateral flexion	Spinous process rotates during lateral flexion
Facet gapping (AP)	C2-7	AP c-spine R and L lateral flexion	Degree of separation at facet during maximal lateral flexion	No appreciable gapping at maximum lateral flexion
Facet gapping (obl)	C2-7	R and L oblique c-spine flexion-extension	Degree of separation at facet during maximal forward flexion	No appreciable gapping at maximum forward flexion
Facet symmetry (obl)	C2-7	R and L oblique c-spine flexion-extension	Degree of symmetrical movement at facets during flexion and extension, comparing right and left	Movement and degree of gapping is symmetrical between sides

Abbreviations: AP = anterior to posterior view, lat = lateral view, obl = oblique view, mm = millimeters, R and L = right and left, SP = spinous process, c-spine = cervical spine.

**Table 2:** Definitions used for each VF parameter measurement.

	Measurements	Dichotomous Recode
C1-2 lateral overhang (AP view)	0–2 mm	0–2 mm > 2 mm
	> 2–4 mm	
	> 4–6 mm	
	> 6 mm	
C1-2 peri-odontoid symmetry (AP view)	Symmetrical	Symmetrical Asymmetrical
	Mildly-Asymmetrical	
	Asymmetrical	
C2-7 translation (lat view)	0–2 mm	0–2 mm > 2 mm
	> 2–3 mm	
	> 3–4 mm	
	> 4 mm	
C2-7 inter-vertebral angulation (lat view)	< 10 degrees	< 10 degrees 10 + degrees
	10 + degrees	
C2-7 SP engagement (lat view)	In sequence	In sequence Not in sequence
	Moderately out of sequence	
	Markedly out of sequence	
C2-7 SP coupled movement (AP view)	In sequence	In sequence Not in sequence
	Moderately out of sequence	
	Markedly out of sequence	
C2-7 Facet gapping (AP view)	Insignificant	Insignificant Not insignificant
	Noticeable	
	Marked	
C2-7 Facet gapping (obl view)	Insignificant	InsignificantNot insignificant
	Noticeable	
	Marked	
C2-7 facet symmetry (obl view)	Symmetrical	Symmetrical Asymmetrical
	Mildly-Asymmetrical	
	Asymmetrical	

Abbreviations: AP = anterior to posterior, lat = lateral, obl = oblique, mm = millimeters, SP = spinous process.

**Table 3:** Crude associations between injured and uninjured groups.

	Symptomatic, n = 119	Asymptomatic, n = 77	*p*-Value *
Demographic (%)			
Female, n (%)	90 (75.6)	36 (46.8)	< 0.0001
Age, mean (se)	40.5 (1.26)	33.9 (1.36)	0.0006
Abnormal VF finding count (%)			
Total abnormal VF findings, mean (se)	1.2 (0.16)	0.13 (0.04)	< 0.0001
C1-C2 lat overhang (AP), n (%)	75 (63.6)	25 (32.5)	< 0.0001
C1-C2 peri-odontoid (AP), n (%)	34 (28.8)	3 (3.9)	< 0.0001
C2-7 translation (lat), n (%)			
C2-C3	38 (31.9)	2 (2.6)	< 0.0001
C3-C4	13 (11.0)	9 (11.7)	0.88
C4-C5	58 (48.7)	19 (24.7)	0.001
C5-C6	23 (19.3)	2 (2.6)	0.001
C6-C7	3 (2.5)	0 (0)	0.16
C2-7 intervertebral angulation (lat), n (%)			
C2-C3	5 (4.2)	0 (0)	0.07
C3-C4	8 (6.7)	1 (1.3)	0.08
C4-C5	30 (25.2)	6 (7.8)	0.002
C5-C6	10 (8.4)	3 (3.9)	0.22
C6-C7	4 (3.4)	0 (0)	0.1
C2-7 SP engagement (lat), n (%)			
C2-C3	2 (1.7)	0 (0)	0.25
C3-C4	15 (12.6)	1 (1.3)	0.005
C4-C5	28 (23.7)	0 (0)	< 0.0001
C5-C6	30 (25.2)	0 (0)	< 0.0001
C6-C7	12 (10.2)	0 (0)	0.004
C2-7 SP coupled motion (AP), n (%)			
C2-C3	3 (2.6)	0 (0)	0.16
C3-C4	11 (9.4)	0 (0)	0.006
C4-C5	32 (27.4)	0 (0)	< 0.0001
C5-C6	49 (41.9)	3 (3.9)	< 0.0001
C6-C7	9 (7.7)	4 (5.2)	0.5
C2-7 facet gapping (AP), n (%)			
C2-C3	1 (0.9)	0 (0)	0.41
C3-C4	14 (12.1)	0 (0)	0.002
C4-C5	40 (34.5)	3 (3.9)	< 0.0001
C5-C6	44 (37.9)	0 (0)	< 0.0001
C6-C7	15 (12.9)	1 (1.3)	0.004
C2-7 facet gapping (obl), n (%)			
C2-C3	3 (2.6)	0 (0)	0.16
C3-C4	14 (12.0)	0 (0)	0.002
C4-C5	56 (47.9)	1 (1.3)	< 0.0001
C5-C6	49 (41.9)	2 (2.6)	< 0.0001
C6-C7	33 (28.2)	2 (2.6)	< 0.0001
C2-7 facet symmetry (obl), n (%)			
C2-C3	3 (2.6)	0 (0)	0.16
C3-C4	10 (8.6)	0 (0)	0.008
C4-C5	32 (27.4)	3 (3.9)	< 0.0001
C5-C6	22 (18.8)	4 (5.2)	0.007
C6-C7	15 (12.9)	1 (1.3)	0.004

* t-test for Age and Total abnormal VF findings; Chi-square for all others. Abbreviations: AP = anterior to posterior view, lat = lateral view, obl = oblique view, mm = millimeters, R and L = right and left, SP = spinous process, c-spine = cervical spine.

**Table 4:** Diagnostic test accuracy results of each of the 3 statistical models.

	Training Data		Testing Data			
	ROC	Lack of Fit *p*-Value	Sensitivity	Specificity	Positive Predictive Value	Negative Predictive Value
Model 1: Stepwise selection *	0.71	-	0.53	1.00	1.00	0.58
Model 2: Number of abnormal readings **	0.94	0.28	0.93	0.89	0.93	0.89
Model 3: Cut point analysis, Number of Abnormal Findings **						
2+	0.88	0.90	0.93	0.79	0.88	0.88
3+	0.92	0.09	0.93	0.84	0.90	0.89
4+	0.92	0.14	0.90	1.00	1.00	0.86
5+	0.90	0.00	0.90	0.68	0.82	0.81

* Model used C4-C5 facet gapping as exemplar abnormal VF parameter ** Adjusted for age and sex. ROC = receiver operating characteristic.

## Supplementary Materials

The following are available online at https://www.mdpi.com/1660-4601/17/5/1693/s1, Table S1: DMX paper analysis data set, Table S2: STARD-2015-Checklist VF study.

## Abbreviations

VF–videofluoroscopy; DMX–digital motion x-ray; PPV–positive predictive value; NPV–negative predictive value; ROC–receiver operating characteristic; CI–confidence interval; MRI–magnetic resonance imaging; CT–computed tomography.

## References

[1] Hoy D.G., Protani M., De R., Buchbinder R. (2010). The epidemiology of neck pain. Best. Pract. Res. Clin. Rheumatol..

[2] Nolet P.S., Emary P.C., Kristman V.L., Murnaghan K., Zeegers M.P., Freeman M.D. Exposure to a Motor Vehicle Collision and the Risk of Future Neck Pain: A Systematic Review and Meta-Analysis. PM&R. http://www.ncbi.nlm.nih.gov/pubmed/31020768.

[3] Insurance Research Council (2008). Auto Injury Insurance Claims: Countrywide Patterns in Treatment, Cost, and Compensation.

[4] McConnell W.E., Howard R.P., Guzman H.M., Bomar J.B., Raddin J.H., Benedict J.V., Smith H.L., Hatsell C.P. (1993). Analysis of Human Test Subject Kinematic Responses to Low Velocity Rear End Impacts. SAE Technical Paper 930889.

[5] Westergren H., Larsson J., Freeman M., Carlsson A., Jöud A., Malmström E.-M. (2018). Sex-based differences in pain distribution in a cohort of patients with persistent post-traumatic neck pain. Disabil. Rehabil..

[6] Ivancic P., Ito S., Tominaga Y., Rubin W., Coe M., Ndu A., Carlson E.J., Panjabi M.M. (2008). Whiplash causes increased laxity of cervical capsular ligament. Clin. Biomech..

[7] Tominaga Y., Ndu A.B., Coe M.P., Valenson A.J., Ivancic P.C., Ito S., Rubin W., Panjabi M.M. (2006). Neck ligament strength is decreased following whiplash trauma. BMC Musculoskelet. Disord..

[8] Steilen D., Hauser R., Woldin B., Sawyer S. (2014). Chronic neck pain: Making the connection between capsular ligament laxity and cervical instability. Open Orthop, J..

[9] Uhrenholt L., Gregersen M., Charles A.V., Hauge E.M., Nielsen E. (2010). Examinations of the deceased can contribute to the understanding of whiplash injuries after traffic accidents. Ugeskr. Laeger..

[10] Cholewicki J., McGill S., Wells R., Vernon H. (1991). Method for measuring vertebral kinematics from videofluoroscopy. Clin. Biomech..

[11] Derrick L.J., Chesworth B.M. (1992). Post-motor vehicle accident alar ligament laxity. J. Orthop. Sports. Phys. Ther..

[12] Krakenes J., Kaale B., Moen G., Nordli H., Gilhus N., Rorvik J. (2002). MRI assessment of the alar ligaments in the late stage of whiplash injury—A study of structural abnormalities and observer agreement. Neuroradiology.

[13] Croft A.C., Krage J., Pate D., Young D. (1994). Videofluoroscopy in cervical spine trauma: An interinterpreter reliability study. J. Manipulative. Physiol. Ther..

[14] Daffner R.H. (2011). Imaging of Vertebral Trauma.

[15] Park M.S., Moon S.-H., Lee H.-M., Kim S.W., Kim T.-H., Suh B.-K., Riew K.D. (2013). The natural history of degenerative spondylolisthesis of the cervical spine with 2- to 7- year follow-up. Spine.

[16] Kopacz K.J., Connolly P.J. (1999). The prevalence of cervical spondylolisthesis. Orthopedics.

[17] Rondinelli R.D., Genovese E., Brigham C.R., American Medical Association (2008). Guides to the Evaluation of Permanent Impairment.

[18] Siegmund G.P., Winkelstein B.A., Ivancic P.C., Svensson M.Y., Vasavada A. (2009). The Anatomy and Biomechanics of Acute and Chronic Whiplash Injury. Traffic. Inj. Prev..

[19] Pettersson K., Hildingsson C., Toolanen G., Fagerlund M., Björnebrink J. (1994). MRI and neurology in acute whiplash trauma: No correlation in prospective examination of 39 cases. Acta Orthop. Scand..

[20] Ferrari R., Kwan O., Russell A.S., Pearce J.M., Schrader H. (1999). The best approach to the problem of whiplash? One ticket to Lithuania, please. Clin. Exp. Rheumatol..

[21] Schmand B., Lindeboom J., Schagen S., Heijt R., Koene T., Hamburger H.L. (1998). Cognitive complaints in patients after whiplash injury: The impact of malingering. J. Neurol. Neurosurg. Psychiatry.

[22] Yoganandan N., Cusick J.F., Pintar F.A., Rao R.D. (2001). Whiplash injury determination with conventional spine imaging and cryomicrotomy. Spine.

